# Supportive treatment of delayed perforated colon due to peritoneal dialysis catheterization

**Published:** 2014

**Authors:** Ahmad Kachoie, Saied Safari, Fatemeh Hosseinzadeh, Jamshid Vafaeimanesh

**Affiliations:** 1 Clinical Research Development Center, Qom University of Medical Sciences, Qom, Iran.

**Keywords:** Peritoneal dialysis, Catheter, Chronic renal failure, Supportive treatment, Bowel perforation.

## Abstract

***Background: ***One of uncommon complications in patients with peritoneal catheter is colon rupture which usually occurs during catheter insertion. In this paper, we present a case of delayed perforated colon following insertion of peritoneal catheter.

***Case presentation:*** A 37-year-old man was suffering from chronic renal failure (CRF). Nine months after peritoneal catheterization, peritoneal dialysis was initiated for him. Dialysis fluid was introduced in the abdomen and severe watery diarrhea appeared. Due to intolerable symptoms (pain and severe watery diarrhea) he was referred to our hospital. By obtaining clinical history and physical examination, with suspicion to probable perforated colon, abdominal radiography with contrast through peritoneal catheter was performed. In his radiography, the catheter was detected in cecum. The patient underwent supportive treatment and the catheter was removed without laparotomy. The symptoms improved with antibiotic therapy, intravenous feeding and initiated bowel rest via NPO (nothing per oral) and he was discharged after 10 days with good general condition.

***Conclusion: ***According to our presentation, it seems that in patients with catheter dysfunction, peritoneal catheter should be immediately removed to prevent colonic perforation.

Today, the number of patients with chronic renal failure (CRF) and consequently, the number of patients who need kidney transplantation and dialysis is increasing. Although the preferential treatment method in these patients is hemodialysis, but peritoneal dialysis is an alternative method for end stage renal disease (ESRD) patients (1). Peritoneal dialysis is considered as a method of treatment in the least developed countries because of its lower costs and not needing expensive hemodialysis centers. The patients under peritoneal dialysis treatment, especially those with partial kidney function, are in suitable clinical condition but they are also in danger of peritoneal dialysis complications. These complications include mechanical and infectious complications. Mechanical complications include bleeding, visceral perforation, catheter dysfunction, dialysate leak, cuff extrusion, hernia formation, and perforated intestinal membrane. Infectious complications include early peritonitis, surgical wound, tunnel and exit site infection (2). In one study, the rate of non-infectious complications of continuous ambulatory peritoneal dialysis (CAPD) was reported 40% and the most common complication was the ultra filtration failure (3). Bowel perforation caused by a peritoneal dialysis catheter occurs very rarely, but has serious consequences (4).

Such perforations mostly occur during catheterization but delayed perforation can also occur some time after catheter insertion (5). 

One of the least common complications of peritoneal dialysis is intestinal perforation which occurs during catheterization process but the delayed bowel perforation due to catheterization was reported but it is more uncommon (6). In this study, we report a case of delayed intestinal perforations due to catheterization which occurred after 9 months of catheterization and peritoneal catheter was not completely used in this period.

## Case presentation

A 37-year-old man with chronic renal failure due to untreated hypertension was adimitted to our hospital. Because of his ESRD, nine months ago, peritoneal dialysis catheter (2-cuffed straight Tenckhoff catheter; Sherwood Medical Company, St. Louis, MO) was implanted into his peritoneal cavity by laparoscopy. He did not undergo peritoneal dialysis within 9 months until presenting uremia symptoms and the peritoneal dialysis was prescribed as the first option. However, the dialysis was impossible due to unsuitable fluid transition. Therefore, second laparoscopy was carried out for the correction of catheter's placement. During laparoscopy, around the catheter was severely fibrosed which had been removed gradually and the catheter was brought to the peritoneum. After two days, the catheter was washed with 100cc normal saline containing heparin and after 10 days, peritoneal dialysis was initiated but it was unsuccessful too.

**Figure 1:**
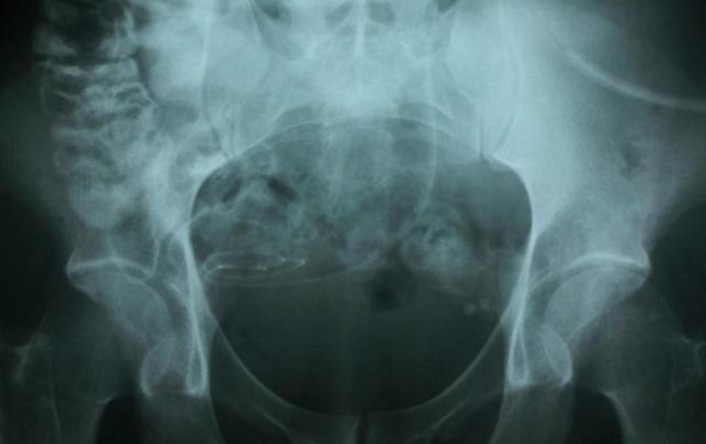
Showing the catheter in the bowel

On admission, he had no fever, abdominal tenderness and signs of peritonitis. Laboratory test showed his blood urea nitrogen (BUN) 78mg/dL, creatinine 4.7mg/dL and according to his weight (67kg), the glomerular filtration rate (BFR) was 20mL/min. After 60 days of correcting placement of catheter, peritoneal dialysis was resumed. After instillation of 500cc dialysis solution into peritoen via catheter, the patient produced severe watery diarrhea and abdominal pain and cramps appeared. Hence, the dialysis was stopped and he was referred to the hospital emergency ward. He was examined by a physician and with suspicion to incorrect placement of catheter, abdominal radiography with water soluble contrast via peritoneal catheter was performed and showed right colon perforation caused by catheter (figure 1).

Supportive treatment was started. Without laparatomy and with the release of proximal and distal cuffs, peritoneal catheter was removed. Oral feeding was stopped and intravenous antibiotic and venous feeding were started. After 10 days of hospitalization, he was discharged with good general condition. No specific pathology was observed during the second laparoscopy.

## Discussion

Colon perforation by peritoneal catheter is one of the rare mechanical complications of this therapeutic method which has been reported rarely in the literature. Most of these patients are older than 60 years and most of them had underlying intestinal pathology (6-10). The reported patient in this study is 37 years old and was the youngest one. 

There was no difference in the prevalence of these complications between laparoscopy and peritoneal catheterization. The presented case is of interest due to the type of treatment which was without laparotomy, patient’s young age, delayed perforation and clinical manifestations like severe watery diarrhea right after the dialysis fluid administration.

The chief complaint in all cases of peritoneal perforation was severe watery diarrhea after peritoneal dialysis. In these cases, intestinal perforation by catheter must be suspected and for certain and precise diagnosis, colonoscopy, contrast fluoroscopy and computed tomography are recommended. After the confirmation of the diagnosis, the patient should be treated (1, 6, 8, 9). Although the treatment of choice in bowel perforations is definitive surgery, but yet the optimal treatment approach has not been established because of the rare prevalence of this complication (6). In reported cases, researchers applied different methods of treatment like supportive treatment including removal of catheter, antibiotic therapy, total venous nutrition, bed rest and hemodialysis (8). The other method was laparoscopic treatment including removal of catheter and closure of perforation by endoscopic clips (6). The last method was invasive treatment, laparotomy, catheter removal and colostomy (10).

We used supportive treatment for our patient and without laparotomy and only with the release of proximal and distal cuff removed the catheter. With broad-spectrum intravenous antibiotic therapy and intravenous fluid administration, the patient was discharged with good general condition. In summary, according to this report several cases of intestinal perforation by catheter it seems that in CRF patients, peritoneal catheterization should be avoided until the need for regular peritoneal dialysis occurs. Also, in patients with catheter dysfunction, peritoneal catheter should be immediately removed to prevent intestinal perforation.
